# Decreased mean perfusion pressure as an independent predictor of acute kidney injury after cardiac surgery

**DOI:** 10.1007/s00380-020-01578-0

**Published:** 2020-03-21

**Authors:** Raymond Hu, Yasmean Kalam, Jeremy Broad, Tim Ho, Frank Parker, Matthew Lee, Rinaldo Bellomo

**Affiliations:** 1 Department of Anesthesia, 145 Studley Road, Austin HealthHeidelberg, VIC 3084 Australia; 2 Department of Intensive Care, 145 Studley Road, Austin HealthHeidelberg, VIC 3084 Australia

**Keywords:** Acute kidney injury, Cardiac surgery, Cardiopulmonary bypass, Central venous pressure, Mean arterial pressure, Mean perfusion pressure

## Abstract

Acute kidney injury after cardiac surgery (AKICS) is common. Previous studies examining the role that mean arterial pressure (MAP) during cardiopulmonary bypass (CPB) may have on AKICS have not taken into account how baseline central venous pressure (CVP) and mean perfusion pressure (MPP) (i.e. MAP − CVP) can influence its evolution. To assess whether the change in MPP to the kidneys (i.e. delta MPP or DMPP) during CPB compared to baseline is an independent predictor of AKICS. After ethical approval, a retrospective observational study was performed on all patients undergoing CPB between October 2013 and June 2015 at a university-affiliated hospital. Known risk factors for the development of AKICS were recorded, as were the MPP values at baseline and during CPB. From this, statistical modelling was performed to identify predictors of postoperative AKICS. 664 patients were identified. Analysis was performed on 513 patients after exclusion. On logistic regression, significant and independent predictors of AKICS included: d20DMPP (cumulative duration of MPP values during CPB that were 20% below baseline and exceeded three consecutive minutes) (*P* = 0.010); baseline CVP; age; pre-operative creatinine level; and left ventricular (LV) dysfunction (ejection fraction (EF) < 45%). On alternative modelling, the cumulative number of MPP values during CPB that were 10% below baseline was also independently associated with AKICS (*P* = 0.003). Modelling without taking into account CVP also supported this association. The duration of differences in perfusion pressure to the kidneys during CPB compared to baseline is an independent predictor of AKICS.

## Introduction

Acute kidney injury (AKI) can be defined by a rise in creatinine of greater than 50% from baseline, according to the RIFLE criteria [1]. In patients who have undergone cardiac surgery, the rate of AKI has been found in up to 30% of patients in high risk populations, with 1–2% of patients requiring renal replacement therapy [2, 3]. AKI after cardiac surgery (AKICS) is associated with increased morbidity, mortality including long-term mortality, and increasing hospital costs [4–7].

Patients who develop AKICS are known to have a higher EuroScore, and be more likely to have valve surgery, emergency surgery or previous cardiac surgery [8, 9]. Nevertheless, these associations do not provide information as to the pathophysiological mechanism for the evolution of AKI. Conversely, careful blood pressure management during cardiopulmonary bypass has been suggested as an important strategy to minimise AKICS [10, 11]. For example, maintaining a mean arterial pressure (MAP) of less than 60 mmHg during cardiopulmonary bypass (CPB) has been associated with renal impairment [10, 12–14]. However, such findings have not been supported by other studies [2, 15–18]. Aiming for an individualized target for CPB MAP that takes into account baseline MAP may be more important than aiming for a particular numerical value [19]. This approach is in line with data in the critical care literature that also suggests that accounting for baseline MAP is important when setting a MAP target to avoid AKI in sepsis [20, 21].

A high central venous pressure (CVP) has also been associated with AKI in the critical care setting and in cardiac surgical patients, which has been attributed to renal congestion [22–25], which would markedly reduce perfusion pressure to the kidneys (MAP − CVP).

Despite the above considerations, previous studies examining the effect of MAP during CPB on AKI in the cardiac surgical population have not accounted for CVP nor for perfusion pressure (MAP − CVP) to the kidneys. Accordingly, we aimed to explore the association between the difference in perfusion pressure to the kidneys during bypass compared to perfusion pressure to the kidneys at baseline on the development of AKICS. This difference has been termed the “Delta Mean Perfusion Pressure” (DMPP). We performed this modelling using DMPP and other factors with the view of ascertaining the particular components of pathophysiology that might be associated with AKICS genesis.

## Materials and methods

### Study design and setting

In a retrospective, observational cohort study, the medical records of patients who underwent cardiopulmonary bypass between October 2013 and June 2015 at our tertiary referral centre were identified. Study approval was granted (LNR/17/Austin/561) and written informed consent was waived.

### Data collection

Intra-arterial MAP values were recorded on a CPB data storage software (Stoeckert S3, Munich Germany), immediately after aortic cannulation and recorded every 20 s until the end of CPB. Baseline hemoglobin values and creatinine values were taken from electronic medical records from the immediate pre-operative visit of each patient (approximately 0–7 days preoperatively). Post-operative serum creatinine was measured at least daily for all patients during their hospital stay. Patient data on comorbidities and vasopressor support were obtained from local Australian and New Zealand Society of Cardiac and Thoracic Surgeons (ANZCTS) database.

We excluded patients if baseline hemodynamic data was unavailable; if AKICS could not be determined [due to death within 24 h, or if the patient had preoperative end stage kidney disease (ESKD)]; continuous systemic arterial pressure during cardiopulmonary bypass was not available (viz*.* any period of circulatory arrest or anterograde cerebral perfusion or missing data during bypass); if the cardiac surgery was not the first operation during their admission; or if cross clamp was not applied (viz*.* pump assist or off-pump cases).

### Patient management

Patients were managed as described by Haase et al*.* [12]. In brief, this involved the same team of cardiologists, surgeons and anesthetists; the cessation of nephrotoxic agents the day before surgery such as non-steroidal anti-inflammatory agents, angiotensin converting enzyme inhibitors, angiotensin II receptor antagonists and diuretics; standardized incision and monitoring; standardized CPB and MAP targets; consistent myocardial perfusion strategy involving blood cardioplegia; and defined postoperative hemodynamic and renal replacement therapy strategies. In particular, target arterial flow intraoperatively was achieved by non-pulsatile CPB flow of 2.4 L/min/m^2^ whilst postoperative cardiac index target was > 2.4 L/min/m^2^ as measured by pulmonary artery catheter. Postoperative MAP targets was > 60 mmHg (or > 70 mmHg in patients with chronic kidney disease, hypertension or otherwise deemed to be at risk of ischemia–reperfusion injury). The use of crystalloids or colloids, and vasopressors was allowed to achieve these targets. Postoperative renal replacement therapy was considered if there was at least one of: urine output < 100 mL for > 6 h unresponsive to fluid resuscitation, potassium > 6.5 mmol/L, pH < 7.2 or clinically significant organ oedema in the setting of renal failure.

Baseline MAP measurements were performed by sphygmomanometry in the holding bay area after routine premedication with opioids (oral oxycodone 10 mg or intramuscular morphine 10 mg) and benzodiazepines (oral diazepam 10 mg or oral lorazepam 1 mg) to remove anxiety as a possible contributor to hypertension, which is our routine practice. Baseline MAP was estimated as diastolic blood pressure + 1/3 times pulse pressure difference. Baseline CVP was taken from the first reading post induction.

### MPP and DMPP and AKI

Baseline mean perfusion pressure (MPP_baseline_) was derived from baseline MAP − baseline CVP. Mean perfusion pressure during bypass (MPP_CPB_) was assumed to equal MAP during bypass, as CVP falls to 0 with venous drainage. Three-minutely median MAP values were obtained during CPB to provide a more robust definition of central tendency, and to mitigate against the effect of transient outliers of MAP values.

DMPP was a priori defined in three separate ways:

- uDMPP (mean DMPP)
  -  = MPP_baseline_ − time weighted MPP_CBP_ values.
- d20DMPP
  -  = cumulative number of median 3-minutely MPP values that are > 20% below MPP_baseline_.
- t20DMPP
  -  = number of times that MPP_CPB_ values are > 20% below MPP_baseline_ (when the preceding value had been < 20% below MPP_baseline_).

AKI after cardiac surgery was defined by the RIFLE criteria, i.e. increase in serum creatinine of greater than 50% from baseline to a peak value within the first seven days, postoperatively [26].

### Statistical method and analysis

Statistical plan consisted of logistic regression modelling using the three definitions of DMPP as the key independent variable and AKICS within 7 days as the outcome, in the presence of other variables.

Model was as outlined by Hosmer et al. [27], while taking into account the limitations set by the number of AKI events and the recorded patient data. For the purpose of building a prediction model, the minimum sample size used was ten AKI events, for each regression coefficient in the logistic regression model. AKI incidence was judged to be at least 15% in this general cohort of cardiac surgical patients. With a plan of 600 records examined, it was anticipated that 90 patients will experience AKI, therefore, the estimated number of regression coefficients that could be utilised was around 9 (including intercept). Additionally, our modelling allowed for confounding and co-linearity as well as the potential for the inability to achieve the minimum number of associated events for each variable.

The following variables were considered for the prediction of AKICS: coefficient of variation of MPP_CPB_, baseline CVP, age, pre-operative creatinine, diabetes, moderate or severe left ventricular (LV) dysfunction (i.e. estimated LVEF < 45%), stroke, New York Heart Association (NYHA) Class III or IV, bypass time, cross clamp time, lowest hemoglobin on bypass, use of intraoperative inotropes, use of ventricular assist device (VAD)/extra corporeal membrane oxygenation (ECMO) and use of intra arterial balloon pump (IABP). These data were obtained from the ANZCTS database according to their data definitions manual (https://anzscts.org/wp-content/uploads/2018/01/ANZSCTS-Data-Definition-Manual-v4.1.pdf) or from the medical records. These variables were reduced following univariate analysis of each potential independent variable and then using best subset selection with models being tested for the strongest fit using the Hosmer–Lemeshow goodness-of-fit test and a likelihood ratio test [27].

Planned testing for robustness of the model included:

- Alternate modelling examining the following variables as covariates in the model instead of the original definitions of DMPP:
  - d10DMPP = cumulative number of median 3-minutely MPP_CBP_ values that are > 10% below MPP_baseline_.
  - t10DMPP = number of times that MPP_CBP_ values are > 10% below MPP_baseline_ (when the preceding value had been < 10% below MPP_baseline_).
- Alternate modelling without accounting for CVP. Note that DMPP is mathematically the same as the difference in MAP on bypass compared to baseline + CVP. That is, delta mean arterial pressure (DMAP) + CVP = DMPP. Therefore, removing CVP from modelling would mean that the following corresponding variables would be tested for significance:
  - uDMAP = MAP_baseline_ − time weighted MAP_CBP_ values.
  - d20DMAP = cumulative number of median 3-minutely MAP_CPB_ values that are > 20% below MAP_baseline_.
  - t20DMAP = number of times that MAP_CBP_ values are > 20% below MAP_baseline_ (when the preceding value had been < 20% below MAP_baseline_).
  - d10DMAP = cumulative number of median 3-minutely MAP_CPB_ values that are > 10% below MAPb_aseline_.
  - t10DMAP = number of times that MAP_CBP_ values are > 10% below MAP_baseline_ (when the preceding value had been < 10% below MAP_baseline_).

All statistical calculations were performed using STATA/SE Version 15.1 software, StatCorp LLC, College Station, TX, USA. The STROBE statement guidelines were followed for reporting of observational studies [28].

## Results

**Fig. 1:**
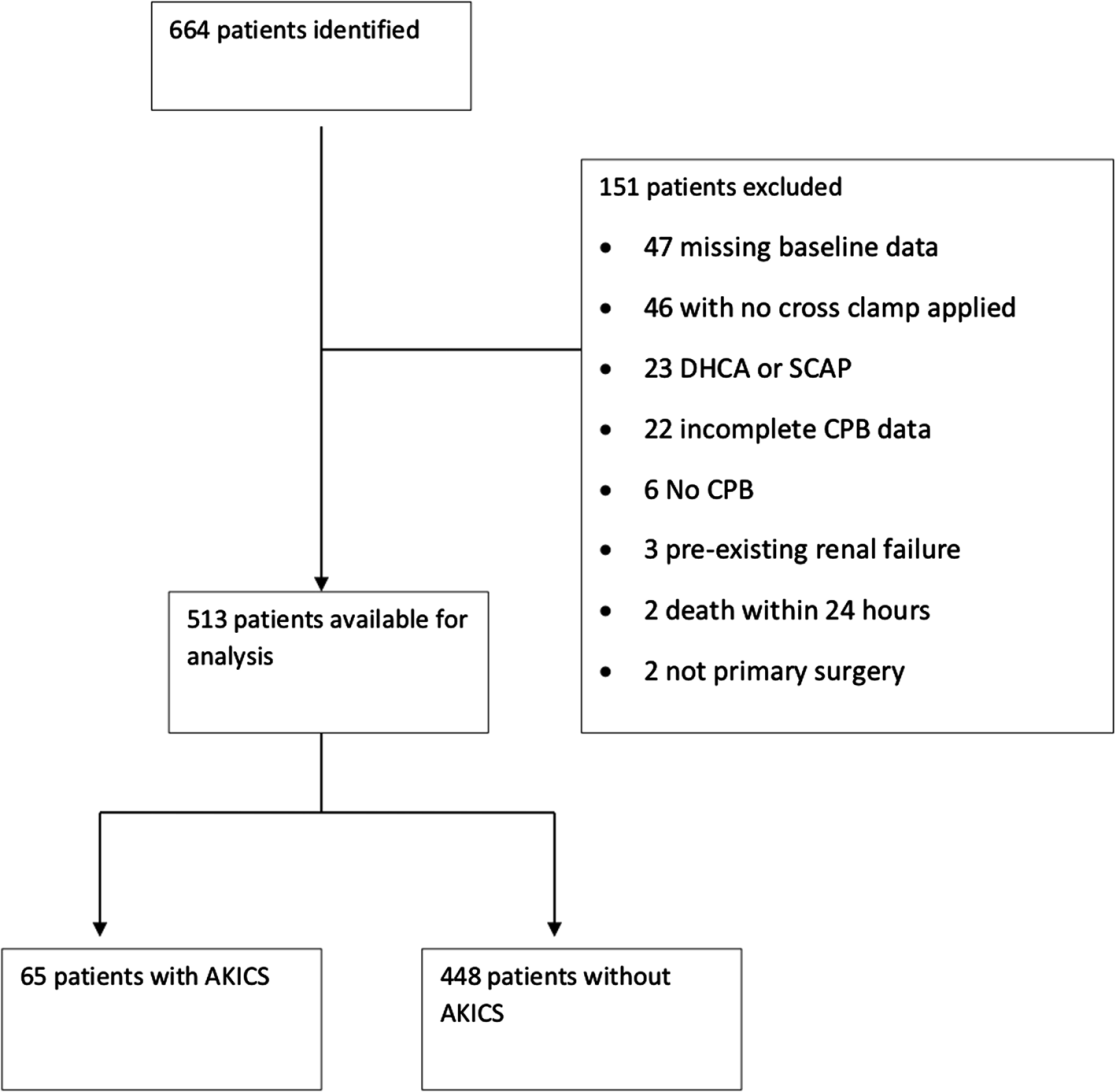
STROBE flow chart. *DHCA *deep hypothermic circulatory arrest, *SCAP *selective cerebral antegrade perfusion, *CPB *cardiopulmonary bypass

Of the 664 records identified, 513 records were used in this model (Fig. 1). Sixty-five patients (12.6%) developed AKI within seven days. Table 1 shows patient demographics and clinical characteristics, prior to CPB and during CPB amongst those who did and did not develop AKI. The variables considered associated with AKICS base on univariate analysis are presented in Table 2 (Model 0).

**Table 1:** Patient characteristics

	All (*n* = 513)		AKI (*n* = 65)		No AKI (*n* = 448)	
	Median (IQR)	Count (%)	Median (IQR)	Count (%)	Median (IQR)	Count (%)
Age (years)*	66 (16.3)		72 (18.0)		66 (16.0)	
Type of surgery						
Any CABG		333 (64.9)		41 (63.1)		292 (65.2)
Any valve surgery*		219 (42.7)		41 (63.1)		178 (39.7)
Any other cardiac surgery		37 (7.2)		6 (9.2)		31 (6.9)
Elective		377 (73.5)		41 (63.1)		336 (75.0)
Emergency*		10 (1.9)		7 (10.8)		3 (0.7)
Urgent		125 (24.4)		16 (24.6)		109 (24.3)
Salvage		1 (0.2)		1 (1.5)		0 (0)
Cardiovascular history						
Hypertension		392 (76.4)		55 (84.6)		337 (75.2)
Atrial fibrillation		80 (15.6)		10 (15.4)		70 (15.6)
Previous MI		175 (34.1)		25 (38.5)		150 (33.5)
Previous cardiac surgery*		19 (3.7)		6 (9.2)		13 (2.9)
Infective endocarditis*		17 (3.3)		7 (10.8)		10 (2.2)
EuroScore*	4 (4.5)		7 (4.5)		4 (4.0)	
LogEuro*	0.032 (0.042)		0.063 (0.096)		0.030 (0.038)	
AusScore*	0.011 (0.014)		0.021 (0.033)		0.010 (0.013)	

*AKI *acute kidney injury, *CABG *coronary artery bypass graft, *MI *myocardial infarction

*P value < 0.05 for AKI vs. No AKI group. The *p* values for differences amongst age, any valve surgery, emergency status, previous cardiac surgery, infective endocarditis, EuroScore, LogEuro and AusScore were: 0.002, 0.001, < 0.001, 0.023, 0.003, < 0.001, < 0.001 and < 0.001, respectively

**Table 2:** Patient variables used in Model 0

Variable	No AKI	AKI	*p* value
uDMPP (mmHg)	21.1 (15.6) [448]	19.4 (17.7) [65]	0.423
d20DMPP (number)	67.4 (49.0) [448]	81.0 (61.0) [65]	0.138
t20DMPP (number)	3.2 (2.1) [448]	3.6 (2.4) [65]	0.214
Coefficient of variation MPP_bypass_	1.4 (0.3) [448]	1.4 (0.3) [65]	0.6180
CVP (mmHg)	8.2 (4.4) [448]	7.8 (5.7) [65]	0.602
Age (years)	63.9 (12.0) [448]	68.6 (12.9) [65]	0.002
Preop creatinine (μmol/L)	89.0 (24.2) [448]	106.7 (52.2) [65]	0.031
Diabetes	132/448	28/65	0.027
Stroke	35/448	9/65	0.166
LV dysfunction	59/448	19/65	0.001
NYHA class III or IV	75/448	20/65	0.010
Lowest hemoglobin on bypass (g/L)	85 (16) [448]	78 (16) [64]	0.003
Bypass time (min)	115 (47) [448]	141 (72) [65]	0.005
Cross clamp time (min)	89 (37) [448]	104 (52) [65]	0.061
Intraoperative inotropes	121/448	30/65	0.003
VAD/ECMO	0/448	2/65	0.016
IABP	3/448	4/65	0.006

Data presented as mean (SD) [*n*] or proportion

*MPP *mean perfusion pressure, *DMPP *delta mean perfusion pressure, *uDMPP *MPP_baseline_ − time weighted average MPP_CBP_, *d20DMPP *cumulative duration that MPP_CBP_ values that were 20% below MPP_baseline_ and exceeded three consecutive minutes, *t20DMPP *number of times that MPP_CBP_ values were more than 20% below MPP_baseline_ and exceeded three consecutive minutes, *CVP *central venous pressure, *LV *left ventricular, *NYHA *New York Heart Association, *VAD *ventricular assist device, *ECMO *extra corporeal membrane oxygenation, *IABP *intra articular balloon pump

### Logistic regression

The final logistic regression model utilised (Model 1) incorporated all variables with *P* values < 0.20 on univariate analysis and also included baseline CVP because its inclusion strengthened the fit of the model with no evidence of collinearity. In this final model, only d20DMPP (cumulative number of median 3-minutely MPP_CPB_ values that were > 20% less than baseline), baseline CVP, age, pre-operative creatinine, and LV dysfunction (EF < 45%) were found to be significant, and independently associated with AKICS (OR > 1, 95% CI). (Table 3).

**Table 3:** Model 1: patient variables independently associated with AKICS (acute kidney injury after cardiac surgery) within 7 days

	Adjusted odds ratio	Standard error	*p* value	95% Confidence interval
d20DMPP (min)	1.006	0.002	0.010	1.002–1.011
CVP (mmHg)	1.064	0.031	0.031	1.006–1.126
Age (years)	1.030	0.013	0.022	1.004–1.057
Preoperative creatinine (μmol/L)	1.011	0.004	0.006	1.003–1.019
LV dysfunction	2.671	0.870	0.003	1.411–5.058

*d20DMPP *cumulative duration that MPP (mean perfusion pressure) values during bypass are > 20% below baseline MPP and exceeded three consecutive minutes, *CVP *central venous pressure, *LV *left ventricle

### Alternative analysis

Alternative analysis using a 10% deviation of mean perfusion pressure from baseline also showed that this variable (d10DMPP) was independently associated with AKICS. Similar associations were found for CVP, age, preoperative creatinine and LV dysfunction (Table 4).

**Table 4:** Alternative modelling for variables independently associated with AKICS within 7 days using DMPP as defined by a > 10% difference in MPP values during bypass compared to baseline

	Adjusted odds ratio	Standard error	*p* value	95% Confidence interval
d10DMPP (min)	1.007	0.002	0.003	1.002–1.012
CVP (mmHg)	1.065	0.031	0.028	1.007–1.128
Age (years)	1.031	0.013	0.018	1.005–1.057
Preop creatinine (μmol/L)	1.011	0.004	0.007	1.003–1.019
LV dysfunction	2.78	0.910	0.002	1.465–5.285

See text for further details

Alternate modelling without accounting for CVP, measuring the difference between MAP_baseline_ and MAP_CPB_ (delta MAP or DMAP) also revealed that d20DMAP (cumulative number of median 3-minutely MAP_CPB_ values were > 20% below MAP_baseline_) and d10MAP (cumulative number of median 3-minutely MAP_CPB_ values that were > 10% below MAP_baseline_) were independently associated with AKICS together with age, preoperative creatinine and LV dysfunction (Tables 5 and 6).

**Table 5:** Alternative modelling for variables independently associated with AKICS within 7 days using DMAP as defined by a > 20% difference in MAP values during bypass compared to baseline

	Adjusted odds ratio	Standard error	*p* value	95% Confidence interval
d20DMAP (min)	1.008	0.002	0.001	1.003–1.012
Age (years)	1.032	0.013	0.010	1.008–1.058
Preop creatinine (μmol/L)	1.011	0.004	0.006	1.003–1.019
LV dysfunction	2.925	0.904	0.001	1.596–5.361

*DMAP *delta mean arterial pressure, *d20DMAP *cumulative duration that MAP (mean arterial pressure) values during bypass are > 20% below baseline MAP and exceeded three consecutive minutes, *LV *left ventricle. See text for further details

**Table 6:** Alternative modelling for variables independently associated with AKICS within 7 days using DMAP as defined by a > 10% difference in MAP values during bypass compared to baseline

	Adjusted odds ratio	Standard error	*p* value	95% Confidence interval
d10DMAP	1.008	0.002	0.000	1.004–1.012
Age	1.034	0.013	0.007	1.009–1.059
Preop creatinine	1.011	0.004	0.005	1.003–1.109
LV dysfunction	2.835	0.874	0.001	1.549–5.186

See text for further details

## Discussion

### Key findings

We performed a retrospective observational study involving 664 cardiac surgery patients receiving cardiopulmonary bypass to assess the independent association between changes in mean perfusion pressure during CPB compared to baseline values and the development of AKICS. On a dataset involving 513 patients, our modelling found that the cumulative number of median 3-minutely values of MPP during bypass that were > 20% from baseline (d20DMPP) was independently associated with the development of AKICS. In particular, every additional minute of d20DMPP was associated with an adjusted odds ratio of 1.006, meaning that if d20MPP was 30, the odds for developing AKICS would be 1.20. Of note, CPB time and cross clamp time did not emerge as independent risk factors after accounting for d20DMPP, which takes into account duration of MPP_CPB_ values that are below baseline. We also found an independent association between CVP, age, pre-operative creatinine and LV dysfunction on the development of AKICS. On alternative analysis, we found that cumulative duration that MPP during bypass that exceeded 10% from baseline and lasted for longer than three consecutive minutes (d10DMP) was also associated with the development of AKICS; furthermore, these relationships remained when CVP was excluded in the modelling. Our study is the first to explore the relationship of DMPP relationship on AKICS. It also confirms the association of baseline CVP as an independent predictor of the development of AKICS.

### Relationship to previous studies

Post-operative AKI occurred in 65 patients (12.6% of patients). This is similar to the incidence of post-operative AKI (3.7–9%) in two previous observational studies investigating AKICS [7, 29]. In our study, patients with a greater pre-operative plasma creatinine level, who were older than 65 years of age, diabetic, with a history of stroke, LV dysfunction or NYHA class III or IV were more likely to have AKI, which is in line with previously documented known non-modifiable risk factors for AKICS [22, 30–35].

As expected, there were differences between those who did and those who did not develop AKICS with regard to their global scores for survival (EuroScore, logEuro, AusScore) as well as the proportion of patients who had valve surgery, emergency surgery, previous cardiac surgery, and infective endocarditis (Table 1). These associations have been noted previously [8, 9]. However, our modelling enabled us to determine the contribution that particular aspects of pathophysiology had towards adverse renal outcomes amongst the entire cohort and our modelling can, therefore, assist with hypothesis generation for therapeutic targets.

Several previous studies have investigated the relationship between MAP during CPB and the incidence of AKI, yielding conflicting results. A study by Haase et al*.* explored the synergistic effect of severe hypotension (MAP < 60 mmHg) and anaemia during CPB. Patients with severe hypotension and anaemia developed AKI more frequently than those patients with severe hypotension [12]. This finding was not corroborated by Sickeler et al*.*, who did not find differences in the rates of AKI between patients who had anaemia with hypotension compared to those patients who only had anaemia [19]. In both these studies however, baseline MAP was not accounted for.

Kanji et al*.* performed a prospective observational cohort study examining the hemodynamic management of patients undergoing CPB for cardiac surgery, investigating the impact of the difference between baseline MAP and the average MAP on CPB on the development of AKI postoperatively [19]. Using multivariate analysis, there was a significant increase in the odds of AKI with a greater difference in MAP. A drop in MAP of greater or equal to 26 mmHg from a preoperative baseline blood pressure was found to have an odds ratio of 2.8 (95% confidence interval 1.3–6.1) for developing CSA-AKI. However, this study did not account for baseline CVP which is increasingly recognized to contribute to AKI.

The influence of CVP on AKI in cardiac patients has been investigated in both medical and surgical settings. In a study by Palomba et al*.* involving the use of an ‘Acute Kidney Injury in Cardiac Surgery’ (AKICS) score to predict AKI in cardiac surgery patients, it was found that low cardiac output and CVP were the only independent hemodynamic risk factors for the development of postoperative AKI [22]. Using univariate regression analysis, as the CVP value approached 14 mmHg postoperatively, there was a two-fold risk in AKI risk [22]. It has also been found that the incidence of AKI is more common in cardiac surgery populations where there is a significant systemic venous congestion, which may be seen in pathological states involving the right heart [36]. Several other studies have found that increased CVP has been associated with worsening renal function with heart failure [23–25].

Mean perfusion pressure (MPP), defined as the difference between MAP and CVP has been explored in the management of septic shock, as an important factor in the development of AKI. Two previous studies involving septic ICU patients, have found that new onset or progressive AKI has been associated with greater MPP deficits compared to those without AKI progression [20, 21].

### Implications of study findings

Our results add support to the notion that the management of mean arterial pressure during bypass is important to mitigate the risk of acute kidney injury after cardiac surgery and needs to consider baseline mean arterial pressure. Additionally, our results suggest that the deleterious effects of a change in mean arterial pressure during bypass from baseline although modest are cumulative. Whilst other risk factors are associated with a higher adjusted odds ratio of developing AKICS (particularly LVEF < 45%), the management of arterial pressure on bypass remains the only modifiable risk factor in the development of acute kidney injury after cardiac surgery. This needs to be investigated in a prospective manner in a wider population. The role of CVP management on renal outcomes also deserves further inquiry and should be investigated in future trials.

### Strengths and limitations

Our study utilised multiple known variables that have been associated with AKICS and has examined the role that hemodynamic management at baseline and during bypass may have on AKICS and is the first study to do so. Our study confirmed associations found in other studies in our univariate analysis, which supports the choice of variables investigated. The verification of our findings with pre-planned alternative assumption analyses also supports the role that hemodynamic management may have on AKICS.

Our study has some notable limitations. First, our planned statistical analysis was based on anticipated high rates of AKI. Additionally, the RIFLE criteria is known to underestimate AKI when compared with the Kidney Disease Improving Global Outcomes (KDIGO) classification [37]. Nevertheless, RIFLE criteria has been extensively used and validated, and our modelling provides sufficient proof of concept to support further investigations examining the effect of both CVP and DMPP on kidney outcomes after cardiac surgery. Furthermore, using a more conservative criteria for AKI in modelling may reduce the risk of Type I error (i.e. establishing a relationship where there is no relationship) which may occur if a less stringent criteria for AKI were used. Several patient groups were excluded from the study including patients with no baseline blood pressure measurements which included many patients undergoing emergency surgery. Additionally, patients undergoing selective anterograde cerebral perfusion or those requiring deep hypothermic circulatory arrest were also excluded. These patients would be expected to have a higher rate of AKICS, and the exclusion of these patients may further explain the low rate of AKI in our study. On the other hand, we included patients who had endocarditis in our analysis which is a group that may have significant confounders in relation to renal outcome to account for a higher rate of AKI (e.g. antibiotic nephrotoxicity). Notwithstanding this, our model can still be useful for hypothesis generation, as has been performed by other investigators who did not exclude patients with endocarditis when investigating AKICS [12].

The measurement of pre-induction blood pressure by sphygmomanometry may not reflect blood pressure at other points of the day or night [38] and may not provide the same value as an invasive reading [39]. However, our cardiac surgical population serves as a unique cohort for modelling baseline blood pressure because of the routine use of anxiolytics for premedication which would be expected to mitigate the effects of stress on blood pressure. Additionally, we recorded blood pressure in the holding bay area which is known to provide a lower value than measurements taken immediately pre-induction [40]. Therefore, the use of this blood pressure as a baseline value in our modelling is still able to provide insights for a pragmatic target for therapy, irrespective of its accuracy as a predictor of “normal” blood pressure at any other point in time before surgery.

Our study was an observational, single centred study, which needs to be replicated in a larger setting. As with all observational studies, confounders may not be accounted for and the potential for bias is possible. An important aspect that was not modelled was the hemodynamics of patients in the pre-bypass period and immediate postoperative period. On the other hand, the potential for collinearity when examining similar variables across different timeframes would have been a limitation with this strategy.

## Conclusions

The cumulative duration of a difference of more than 20% between mean perfusion pressure to the kidneys during cardiopulmonary bypass compared to mean perfusion pressure to the kidneys at baseline is an independent and modifiable predictor of postoperative AKI. This effect was also seen at more modest deviations in perfusion pressure and when CVP values were not known. These observations suggest the need to conduct randomized trials comparing different blood pressure targets during cardiopulmonary bypass relative to baseline values to determine whether limiting the duration of differences can safely prevent or attenuate post-operative AKI.
